# Merging Children’s Oncology Group Data with an External Administrative Database Using Indirect Patient Identifiers: A Report from the Children’s Oncology Group

**DOI:** 10.1371/journal.pone.0143480

**Published:** 2015-11-25

**Authors:** Yimei Li, Matt Hall, Brian T. Fisher, Alix E. Seif, Yuan-Shung Huang, Rochelle Bagatell, Kelly D. Getz, Todd A. Alonzo, Robert B. Gerbing, Lillian Sung, Peter C. Adamson, Alan Gamis, Richard Aplenc

**Affiliations:** 1 Department of Biostatistics and Epidemiology, University of Pennsylvania Perelman School of Medicine, Philadelphia, Pennsylvania, United States of America; 2 Division of Oncology, Children’s Hospital of Philadelphia, Philadelphia, Pennsylvania, United States of America; 3 Children’s Hospital Association, Overland Park, Kansas, United States of America; 4 Division of Infectious Disease, Children’s Hospital of Philadelphia, Philadelphia, Pennsylvania, United States of America; 5 Center for Pediatric Clinical Effectiveness, Children’s Hospital of Philadelphia, Philadelphia, Pennsylvania, United States of America; 6 Department of Preventive Medicine, University of Southern California, Los Angeles, California, United States of America; 7 Children’s Oncology Group, Monrovia, California, United States of America; 8 Department of Hematology/Oncology, The Hospital for Sick Children, University of Toronto, Toronto, Canada; 9 Division of Hematology/Oncology/Bone Marrow Transplantation, Children’s Mercy Hospital and Clinics, Kansas City, Missouri, United States of America; University of Arkansas for Medical Sciences, UNITED STATES

## Abstract

**Purpose:**

Clinical trials data from National Cancer Institute (NCI)-funded cooperative oncology group trials could be enhanced by merging with external data sources. Merging without direct patient identifiers would provide additional patient privacy protections. We sought to develop and validate a matching algorithm that uses only indirect patient identifiers.

**Methods:**

We merged the data from two Phase III Children’s Oncology Group (COG) trials for *de novo* acute myeloid leukemia (AML) with the Pediatric Health Information Systems (PHIS). We developed a stepwise matching algorithm that used indirect identifiers including treatment site, gender, birth year, birth month, enrollment year and enrollment month. Results from the stepwise algorithm were compared against the direct merge method that used date of birth, treatment site, and gender. The indirect merge algorithm was developed on AAML0531 and validated on AAML1031.

**Results:**

Of 415 patients enrolled on the AAML0531 trial at PHIS centers, we successfully matched 378 (91.1%) patients using the indirect stepwise algorithm. Comparison to the direct merge result suggested that 362 (95.7%) matches identified by the indirect merge algorithm were concordant with the direct merge result. When validating the indirect stepwise algorithm using the AAML1031 trial, we successfully matched 157 out of 165 patients (95.2%) and 150 (95.5%) of the indirectly merged matches were concordant with the directly merged matches.

**Conclusions:**

These data demonstrate that patients enrolled on COG clinical trials can be successfully merged with PHIS administrative data using a stepwise algorithm based on indirect patient identifiers. The merged data sets can be used as a platform for comparative effectiveness and cost effectiveness studies.

## Introduction

National Cancer Institute (NCI)-funded cooperative group clinical trials have improved cure rates for children with cancer and have set standards of care for the treatment of adult malignancies [1]. However, such clinical trials data have important limitations, particularly the lack of resource utilization and cost data. Administrative data sets contain detailed resource utilization data that can be used to describe supportive care practices and treatment costs, but often lack accurate disease-specific information such as pathology validated diagnosis, risk stratification data, and disease recurrence. Linking the NCI funded cooperative group data with external administrative data sets would enable a wide range of comparative effectiveness and cost effectiveness studies.

Other investigators have previously merged data from adult cooperative oncology groups with Medicare claims data [2–4]. Our group has merged pediatric data from a Children’s Oncology Group (COG) phase III trial with the administrative data from the Pediatric Health Information Systems (PHIS) [5]. In our previous work, we used the direct patient identifier date of birth (DOB), treatment site, and gender to link records across the two datasets. The use of DOB in the merging process raises privacy concerns and necessitates human subject research permission from regulatory bodies. Merging without the use of DOB would substantively alleviate privacy concerns and lessen regulatory requirements. Therefore the objective of this study was to develop and validate such a matching algorithm to link the COG clinical trial data and the PHIS administrative data using only indirect patient identifiers.

## Methods

### Data sources

COG is the pediatric cooperative oncology group funded by the NCI and has approximately 200 actively participating centers in the United States, Canada, Europe, and Australia. AAML0531 was a randomized clinical trial to compare standard chemotherapy with or without gemtuzumab for the treatment of *de novo* acute myeloid leukemia (AML) and enrolled 1,022 eligible patients from August 14, 2006 to June 15, 2010 [6]. AAML1031 is a randomized trial to compare standard chemotherapy with or without bortezomib for the treatment of *de novo* AML. The enrollment of AAML1031 is ongoing and had enrolled 449 patients by the time of this analysis. Like all COG therapeutic trials, the AAML0531 and AAML1031trials collected extensive data on leukemia phenotype, demographics, clinical data such as central nervous system involvement, and clinical outcomes including mortality, leukemia relapse, second malignancy, toxicity and bone marrow transplant status.

PHIS is an administrative database including data from 44 free-standing pediatric hospitals in the United States that are affiliated with the Children’s Hospital Association (CHA; Overland Park, KS; data management center). These hospitals represent most of the major metropolitan areas in the U.S. PHIS data have previously been used in over 300 peer-reviewed publications including studies of patients with AML from our group [7–13]. Oversight of PHIS data quality methods is a joint effort between CHA, Truven Health Analytics (data processing partner, Ann Arbor, MI), and participating hospitals. Each hospital submits its data to Truven Health Analytics quarterly, and data quality audits are performed (e.g., check for valid ICD‐9‐CM diagnosis codes and reasonable patient information such as birth weight). Member hospitals have access to the PHIS database through a secure web-based reporting system.

PHIS data are composed of two levels: Level 1 data come from the hospital’s medical record system and include patient identification (encrypted medical record number), demographics, dates of admission and discharge, payer information and ICD-9 diagnosis and procedure codes (up to 41 codes per admission); Level 2 data come from the hospital’s billing system and include pharmaceuticals and blood products ordered, imaging requested, and clinical services utilized, with a date on which they were ordered and their route of administration. Cost data is also estimable from charges using hospital-specific cost-to-charge ratio for the relevant department. Table 1 compares the data elements available from both COG and PHIS data sources.

**Table 1:** Data elements available in COG and PHIS.

Variables	COG	PHIS
Demographics	Yes	Yes
Diagnosis	Yes	Yes (Inferred from ICD-9)
Risk stratification data	Yes	No
Biological specimen	Yes	No
Mortality	Yes	Yes (Inpatient)
Leukemia relapse status	Yes	Yes (Inferred from ICD-9)
Bone marrow transplant status	Yes	Yes (Inferred from ICD-9)
Toxicities	Yes (Reported by Data Managers)	Yes (Inferred from ICD-9 and Resource Data)
Medications	No	Yes
Procedures	No	Yes
Blood bank resources	No	Yes
Radiology services/procedures	No	Yes
Cost data	No	Yes (PHIS adjusted charge and cost-to-charge ratio)

### Development and validation of the merge algorithm

The COG data set in this study was comprised of enrolled eligible patients on AAML0531 or AAML1031 and included DOB, treatment site, enrollment date, gender, race and insurance status. COG enrollment date was replaced by the transfer date for those patients who transferred to a PHIS center from another hospital. The PHIS data set was inclusive of patients with an AML ICD-9 code (ICD-9 codes for AML or unspecified leukemia: 205.xx, 206.xx, 207.xx, or 208.xx) admitted during the time period that each trial was open. The PHIS data set included DOB, treatment site, admission start date, gender, race and insurance status.

Subsequently, COG and PHIS data were merged in a multistep process. First, COG data for AAML0531 patients only was used to develop and optimize the indirect algorithm to merge with PHIS data. Next, the optimal indirect merge algorithm was performed using COG AAML1031 patients to validate the process. The indirect merge algorithm consider the following data elements: treatment site, gender, birth year, birth month, COG enrollment/PHIS admission year, and COG enrollment/PHIS admission month (Table 2). The algorithm contained four steps; patients who found a unique match in one step were not included in the subsequent matching steps. The algorithm started with the most rigorous criterion that required patients to be matched on all six variables (step 1), and then removed enrollment/admission month in step 2 and enrollment/admission year in step 3. The last step added back the enrollment/admission year but allowed this variable to be different by +/-1 from the two data sets.

**Table 2:** The stepwise merge algorithm using indirect patient identifiers.

	Step			
Matching variables	1	2	3	4
Treatment site	=	=	=	=
Gender	=	=	=	=
Birth year	=	=	=	=
Birth month	=	=	=	=
Enrollment/admission year	=	=		+/- 1
Enrollment/admission month	=			

We also merged the COG and PHIS data sets using the direct merge method, which used DOB, treatment site, and gender. Results from the indirect and direct merge were compared.

### Analysis

In each of the four steps of the indirect stepwise algorithm, we summarized the number and proportion of patients with (a) a unique match, (b) no match, and (c) multiple matches. The cumulative percent of patients with a unique match was also calculated. In the comparison of the indirect and direct methods, a unique match identified by the indirect stepwise algorithm was considered concordant with the direct merge if it was the same as the unique match from the direct merge method. If the indirect algorithm yielded a unique match but the direct merge method yielded duplicate matches, we initially considered this as a discordant match (Criterion 1). However, in a second evaluation, we considered this a concordant match if the match in the indirect merge method was among one of the duplicate matches in the direct merge method (Criterion 2). We summarized the number and proportion of concordant matches among the unique matches for each step of the indirect algorithm and cumulatively.

### Protection of Human Subjects

All patients enrolled on AAML0531 and AAML1031 gave informed consent for use of clinical trial data for research. All patient data remained de-identified throughout the merging process. AAML0531, AAML1031, and the merging study were approved by the Institutional Review Board at the Children’s Hospital of Philadelphia.

## Results

Of 1,022 eligible patients enrolled on AAML0531, 415 (40.6%) were treated at institutions contributing to PHIS. Table 3 presents the results of the derived indirect stepwise algorithm. In step 1 only 204 (49.2%) unique matches were achieved. The inability to uniquely match the remaining patients was primarily due to discrepancies in the enrollment/admission month and year from the two data sources. Therefore by removing enrollment/admission month in step 2 and enrollment/admission year in step3, we were able to identify an additional 128 and 33 unique matches, respectively. With fewer matching constraints in steps 2 and 3, there were more COG patients matched to multiple records from PHIS. By step 3 there were 34 COG patients matched with multiple PHIS records. In an effort to reduce these multiple match scenarios, enrollment/admission year was added back in step 4 allowing for a window of +/-1 year. This identified 13 additional unique matches. The cumulative percent of unique matches for the indirect stepwise algorithm was 91.1% (378 out of 415 patients). In contrast, the percent of unique match for the direct merge method was 92.3% (383 out of 415 patients). As described in [5], using the direct merge method on AAML0531, patients who were matched and patients who were not matched had similar demographics including age, gender and race, so the matched patients were a representative sample of the entire trial population. In addition to the initial admission, the matching rates for subsequent courses of chemotherapy were also high (92% to 95%) [5].

**Table 3:** Matching results using the indirect stepwise algorithm, developed on AAML0531. Note: The number of unique match from the direct merge method was 383 (92.3%).

		Step 1	Step 2	Step 3	Step 4
	Number of patients available for match	415	211	83	50
	Number of patients with a unique match (%)	204 (49.2%)	128 (60.7%)	33 (39.8%)	13 (26.0%)
	Number of patients with no match (%)	204 (49.2%)	66 (31.2%)	16 (19.3%)	16 (32.0%)
	Number of patients matched with multiple PHIS records (%)	5 (1.2%)	15 (7.0%)	34 (40.9%)	21 (42.0%)
	Number of patients matched with multiple COG records (%)	2 (0.4%)	2 (0.9%)	0 (0%)	0 (0%)
	Cumulative number of unique matches (%)	204 (49.2%)	332 (80.0%)	365 (88.0%)	**378 (91.1%)**
Criterion 1*					
	Number of unique matches that are concordant with the direct method (%)	198 (97.1%)	121 (94.5%)	33 (100%)	10 (76.9%)
	Cumulative number of unique matches that are concordant with the direct method (%)	198 (52.4%)	319 (84.4%)	352 (93.1%)	**362 (95.7%)**
Criterion 2**					
	Number of unique matches that are concordant with the direct method (%)	202 (99.0%)	122 (95.3%)	33 (100%)	12 (92.3%)
	Cumulative number of unique matches that are concordant with the direct method (%)	202 (53.4%)	324 (85.7%)	357 (94.4%)	**369 (97.6%)**

* Criterion 1 considers a match as discordant, if the indirect algorithm yielded a unique match but the direct merge method yielded duplicate matches.

** Criterion 2 considers a match as concordant, if the indirect algorithm yielded a unique match but the direct merge method yielded duplicate matches, and the match in the indirect merge method was among one of the duplicate matches in the direct merge method.

We then compared the performance of the indirect merge algorithm against the direct merge method (Table 3). Using Criterion 1, among the 204 unique matches in step 1, 198 (97%) were concordant. The percent of concordant matches ranged from 76.9% to 100% across the four matching steps, and the cumulative percent of concordant matches was 95.7% (362 out of 378). Using Criterion 2, the percent of concordant matches ranged from 92.3% to 100% across the four matching steps, and the cumulative percent of concordant matches was 97.6% (369 out of 378).

Table 4 presents the results of the indirect stepwise algorithm when we used the AAML1031 trial data as a validation. The results were similar to what we observed in AAML0531. Among the COG AAML1031 cohort, 165 patients were treated at PHIS centers. The indirect merge algorithm identified 129 unique matches in step 1, 22 in step 2, and six in step 3. Therefore, the cumulative percent of patients with a unique match was 95.2% (157 out of 165 patients). This was slightly better than the matching rate of the direct merge method (91.5%, 151 out of 165 patients), because the indirect algorithm was able to find a unique match for some patients who were matched with duplicates in the direct method. When comparing the unique matches from the indirect algorithm to the direct merge result, we found that 150 (95.5%) matches were concordant based Criterion 1 and 155 (98.7%) matches were concordant based on Criterion 2.

**Table 4:** Matching results using the indirect stepwise algorithm, validated on AAML1031. Note: The number of unique match from the direct merge method was 151 (91.5%).

		Step 1	Step 2	Step 3	Step 4
	Number of patients available for match	165	36	14	8
	Number of patients with a unique match (%)	129 (78.2%)	22 (61.1%)	6 (42.9%)	0 (0%)
	Number of patients with no match (%)	30 (18.2%)	7 (19.4%)	0 (0%)	1 (12.5%)
	Number of patients matched with multiple PHIS (%)	6 (3.6%)	7 (19.4%)	8 (57.1%)	7 (87.5%)
	Number of patients matched with multiple COG records (%)	0 (0%)	0 (0%)	0 (0%)	0 (0%)
	Cumulative number of unique matches (%)	129 (78.2%)	151 (91.5%)	157 (95.2%)	**157 (95.2%)**
Criterion 1 *					
	Number of unique matches that are concordant with the direct method (%)	124 (96.1%)	22 (100%)	4 (66.7%)	0 (NA)
	Cumulative number of unique matches that are concordant with the direct method (%)	124 (79.0%)	146 (93.0%)	150 (95.5%)	**150 (95.5%)**
Criterion 2 **					
	Number of unique matches that are concordant with the direct method (%)	129 (100%)	22 (100%)	4 (66.7%)	0 (NA)
	Cumulative number of unique matches that are concordant with the direct method (%)	129 (82.2%)	151 (96.2%)	155 (98.7%)	**155 (98.7%)**

* Criterion 1 considers a match as discordant, if the indirect algorithm yielded a unique match but the direct merge method yielded duplicate matches.

** Criterion 2 considers a match as concordant, if the indirect algorithm yielded a unique match but the direct merge method yielded duplicate matches, and the match in the indirect merge method was among one of the duplicate matches in the direct merge method.

## Discussion

We have developed a stepwise matching algorithm to merge COG clinical trial data with the PHIS administrative database using only indirect patient identifiers. Our results show that the matching rate of the derived algorithm using the AAML0531 cohort is high (>91%) and comparable to that of the direct merge method, and the vast majority of the algorithm-unique matches were concordant with the direct merge-unique matches. The indirect merge algorithm was then validated using a second COG clinical trial data set, AAML1031.

The use of indirect patient identifiers to link different datasets has been described in studies of various diseases [14–18]. Newgard linked ambulance records to a state trauma registry with a matching rate of 96% and no validation of the matches [14]; Meray et al linked three Dutch Perinatal Registries, had a matching rate of 66% and in a subsample validation >99% of the matched pairs were validated [15]; Hammill et al linked inpatient clinical registry data to Medicare claims data with a matching rate of 91% and no validation [16]; Pasquali et al linked a heart surgery clinical registry data to PHIS, had a matching rate of 90% and in a subsample validation 100% of the matches were validated [17]; Lawson et al linked a clinical surgical registry to Medicare inpatient claims data with a matching rate of 81% and no validation [18]. However, to our knowledge, our study is the first attempt at exploring this methodology to merge two data sources for pediatric oncology patients, with one data source from cooperative group oncology clinical trials. The derivation of this matching algorithm found that a multi-step procedure was necessary, as a one-step matching process did not achieve a high rate of unique matches. In addition to deriving the indirect merge we were able to confirm its success in a separate COG cohort with similar matching success. Compared to other studies, our study had similar or higher matching rates (91%–96%) and all the matches were validated against a direct matching method with >95% concordance rates.

Our study has some limitations. First, the indirect merge algorithm failed to find a match for some patients (<4%). Examination of the unmatched patients did not reveal any patterns of patient characteristics influencing matching success. The primary reason of not identifying a match was the discrepancy between COG enrollment year/month and PHIS admission year/month and such discrepancies seemed to stem from random errors in the PHIS admission year/month data. Second, the indirect merge algorithm matched some COG records with multiple PHIS records (<5%). This multiple matching occurred because variables in the algorithm were not unique enough to differentiate all the patients. We attempted to resolve this issue by including more matching variables such as insurance and race. However these variables were coded differently in the two databases and, although we created a crosswalk to match different categories of these variables, they were not reliable enough to further improve matching rates. Therefore the algorithm in its current format would perform less reliably with more prevalent conditions such as more common cancers that occur in adults, as there would likely be many patients with the same combination of the identifiers. In those settings, our method would need to be adapted, for example, by incorporating information on the subsequent admissions into the matching process. Third, we did not have a gold standard (i.e. primary chart review) to prove that unique matches from the indirect or direct merges were indeed accurate. However, the majority (>95%) of the unique-matches from the indirect merge for both COG trials were concordant with the unique-matches from the direct methods. This concordance suggests that cohorts comprised of uniquely matched patients from the indirect algorithm would be appropriate for inclusion in further analyses.

This merge was done using data from the first chemotherapy course of AML treatment, but once a patient is matched, they can be successfully followed for subsequent courses in PHIS [5]. These merged data sets provide opportunities for comparative effectiveness and cost effectiveness research efforts that are ongoing.

Although the indirect matching algorithm was established in COG AML trials, this merging procedure should be generalizable to COG trials in other pediatric malignancies. Because the algorithm only utilized basic demographic variables, we expect that merging results in other COG trials will have a comparable success rate. Work is ongoing to merge PHIS database with COG trials for acute lymphoblastic leukemia and neuroblastoma. Ultimately, these merged data sets will serve as a research platform that enables investigators to address important clinical epidemiology research questions for pediatric cancers.
